# Factors Influencing Behaviors to Reduce the Spread of COVID-19 Among Indigenous Families in the Southwest, United States

**DOI:** 10.3390/ijerph21111407

**Published:** 2024-10-24

**Authors:** Habibat A. Oguntade, Miya Pontes, Karlita Pablo, Elliott Pablo, Novalene Goklish, Meredith Stifter, Lauren Tingey, Allison Barlow, Laura L. Hammitt, Mary Cwik

**Affiliations:** 1 Department of International Health, Johns Hopkins Bloomberg School of Public Health, Baltimore, MD 21205, USA; 2 Division of Epidemiology & Community Health, School of Public Health, University of Minnesota, Minneapolis, MN 55454, USA; 3 Department of Psychiatry and Behavioral Sciences, Johns Hopkins School of Medicine, Baltimore, MD 21205, USA

**Keywords:** American Indian and Alaska Native, Indigenous, barriers and facilitators, COVID-19 information, COVID-19 testing, COVID-19 vaccination, mask-wearing, social distancing

## Abstract

Indigenous communities in the United States (U.S.) have been disproportionately impacted by COVID-19, yet they have led efforts to combat the pandemic by providing local solutions that minimize viral transmission and promote vaccine uptake. Understanding facilitators and barriers to recommended behaviors can increase adherence and reduce COVID-19 transmission. We conducted a descriptive qualitative study using in-depth interviews with 19 Indigenous adults residing on Tribal Lands in the Southwest U.S. between June and December 2021. Interview questions explored obstacles and motivators to testing, as well as behavioral recommendations to reduce COVID-19 transmission and increase vaccination. Using a qualitative content analysis approach, we identified barriers and facilitators to behavioral change. Barriers to testing included fear of exposure to COVID-19 at testing locations and discomfort from nasal swabs. Facilitators of testing were access, required testing, and protecting loved ones. Barriers to vaccination were discouraging stories about vaccination side effects and uncertainty about the ingredients and effectiveness of vaccines. Another barrier to vaccination was confusion and fairness related to vaccine eligibility, which discouraged some people from becoming vaccinated, despite intentions to do so. This study identified obstacles and motivators influencing COVID-19 testing and vaccination. The results may help address information gaps and improve public health efforts to reduce COVID-19 transmission and other similar infectious agents in Indigenous communities.

## 1. Introduction

The emergence of the novel coronavirus, SARS-CoV-2, and the global COVID-19 pandemic in March 2020, precipitated widespread efforts to minimize viral transmission [1,2,3]. Isolating symptomatic individuals alone proved inadequate in controlling its transmission, prompting the implementation of lockdowns and social distancing guidelines. The pandemic had a disproportionate impact on Indigenous communities, with alarming rates of infection and mortality [4,5]. Beyond the immediate health impact, the pandemic highlighted the significant role of social determinants in perpetuating health inequities in Indigenous populations [6]. Past respiratory viral pandemics have shown substantially higher death rates among Indigenous communities compared to the general U.S. population, underscoring the need to address broader systemic issues [7]. Factors such as food and water insecurity, exposure to environmental toxins, and historical barriers to healthcare access contribute to the higher burden of disease in these communities [8]. Additionally, socioeconomic challenges experienced by Indigenous communities, including lack of running water [9] and overcrowded housing, have further exacerbated their susceptibility to infection.

More broadly, the pandemic caused acute psychosocial stress, leading to an increase in mental health issues like substance abuse, depression, and anxiety. These mental health challenges further contributed to the pandemic’s spread. In all communities, including Indigenous communities, people might have shared substances more frequently, avoided testing due to stigmas associated with testing positive, or become trapped in ongoing cycles of fear and despair [10]. The COVID-19 pandemic was also a profound reminder of the injustices endured by Indigenous populations, simultaneously inciting trauma and demanding tribal resilience to survive and thrive anew. Despite the generational trauma, persistent inequities, and health systems challenges Indigenous peoples have faced [11], these communities also led the United States in pursuing lifesaving behaviors to prevent the spread of COVID-19.

American Indian and Alaska Native (AI/AN) peoples held the highest percentages of people receiving the first dose and full vaccination of the COVID-19 vaccine compared with other US races [12]. This high uptake of vaccination demonstrates a collective dedication to protecting the health of community members [12]. This accomplishment has been widely recognized and serves as a model for other communities.

AI/AN communities saw both the incredible uptake of lifesaving behaviors, and the devastating impacts of COVID-19, including greater rates of mortality, intubation, and ICU admission [13]. In this context, this study sought to examine the factors that influence behaviors like COVID-19 testing and subsequent vaccine acceptance within high-risk Indigenous populations. The objective was to leverage findings to enhance future pandemic readiness, particularly concerning testing and immunization outreach, as well as to optimize logistical assistance, thereby mitigating potential disparities in health equity. Furthermore, the insights gained will contribute to shaping future public health initiatives aimed at preventing inequity gaps among Indigenous communities during future pandemics or health crises.

## 2. Materials and Methods

### 2.1. Study Design and Setting

This descriptive qualitative study was nested within a larger randomized controlled trial (RCT)—Protecting Native Families from COVID-19 (PROTECT; 3U19MH113136-04S2). The PROTECT study has been described elsewhere [14]. Briefly, PROTECT is an RCT where longtime tribal-academic research partners, the Johns Hopkins University (JHU) Center for Indigenous Health (CIH), White Mountain Apache Tribe (WMAT), and Navajo Nation, aimed to evaluate a culturally tailored motivational interviewing intervention (MI) and a text-based COVID-19 daily symptom checker (CS) to increase facilitators and decrease barriers to diagnostic testing and preventative behaviors among two high-risk groups in these communities—young adults, who contribute significantly to the spread of COVID-19, and Elders, who suffer more severe impacts from the virus. The overall goal of the PROTECT study was to increase testing among those with COVID-19 symptoms and improve adherence to recommended strategies to reduce the spread of COVID-19 following positive test results in these high-risk groups. One of the secondary aims of PROTECT, highlighted in this paper, was to explore barriers and facilitators to testing and vaccine uptake among study participants to inform future public health efforts. To achieve this aim, we conducted a descriptive qualitative study [15], utilizing purposive sampling to select a subset of 19 PROTECT participants for in-depth interviews between June and December 2021. The reporting of our study methodology follows the consolidated criteria for reporting qualitative research (COREQ) [16].

This qualitative sub-study was conducted with PROTECT participants residing in White Mountain Apache Tribal Lands. The most recent population estimate for the White Mountain Apache Tribal Lands was 14,854, according to the U.S. Census [17]. The first COVID-19 case was documented April 2020, and by 15 February 2021, there were 3904 cases of COVID-19 [18]. Compounding the situation, the prevalence of underlying conditions, such as obesity, heart disease, and diabetes, was high, which further amplified vulnerability to severe COVID-19 [19].

### 2.2. Ethical Considerations

The study was approved by the JHU Institutional Review Board, White Mountain Apache Tribal Council and Health Board and Phoenix Area Indian Health Service Institutional Review Board.

### 2.3. Participants

Initially, the PROTECT study planned to enroll eligible participants based on the following criteria: young adults aged 18–34 with a recent history of substance use problems OR elders ≥ 55 who identified as American Indian (AI). The study was conducted in Whiteriver, AZ; Chinle, AZ; and Shiprock, NM, and participants needed to reside within a 60-mile radius of the respective hospital at each site. As the COVID-19 pandemic’s situation evolved, the study’s protocols were adapted in response to community partner feedback, particularly concerning vaccine hesitancy. This feedback was elicited from the PROTECT Community Advisory Board (CAB) members and local JHU staff who identified as members of Indigenous communities. Consequently, vaccinated tribal members became ineligible for PROTECT; therefore, only those who were unvaccinated participated in the interviews. These protocol amendments resulted in the enrollment of only 64 participants: 38 from WMAT and 26 from NN. Despite initial intentions to draw participants from all three designated PROTECT sites for the qualitative study, the final participant pool was exclusively composed of individuals from the WMAT community, mirroring the primary demographic of PROTECT participants.

Participants were recruited through radio, print, and social media advertisements, through existing health clinic visits for non-COVID-19 related services, and through ongoing contact tracing and Indian Health Service (IHS) referrals. Recruitment flyers were designed by a local artist from the WMAT, and all advertisements provided the phone number of the participating JHU office. This enabled potential participants to learn about the study’s details, determine eligibility, and ask questions about the study. These conversations were led by trained JHU Research Program Assistants, who are also tribal members and were guided by a standard recruitment script. Trained Research Program Assistants obtained informed consent from participants prior to enrolling in PROTECT. The consent discussion was guided by a consent flipchart that clearly outlined all information in the consent form. Additionally, participants were informed that they could be invited to join a qualitative study upon completing all four study assessments. Participants were given a consent comprehension quiz following the consent discussion. For the qualitative study, a purposive sampling approach was used to select PROTECT participants who had completed final study assessments and who were accessible, responsive, and more likely to provide rich information when recruited for the qualitative study. All the participants had also been involved in other aspects of the larger PROTECT study, which allowed the study staff to become familiar with them and better assess their suitability for the qualitative study. Program assistants regularly reviewed study enrollment logs and conducted weekly calls to assess participants’ responsiveness, communication styles, and prior engagement with the study to identify those who were more likely to provide rich, detailed information.

### 2.4. Data Collection

Data collection for the qualitative study took place from June to December 2021. The interviews were conducted using a semi-structured interview guide developed by the PROTECT team. The guide was designed to be administered through one-on-one in-person interviews by a single interviewer. It consisted of open-ended questions aimed at assessing participants’ experiences with COVID-19 testing, vaccination, and recommended guidelines to prevent the spread of COVID-19. Examples of questions in the interview guide are: “*How likely are you to get tested in the next month? Why? What factors did you consider when coming to this decision?*”, “*When told positive or negative things about the COVID-19 vaccine, do you research information for yourself? If so, who/what do you consider trustworthy sources of information?*”, and “*What could convince you to start/remain socially distant from others?*” Additionally, participants were asked a series of questions about their experiences participating in the PROTECT study. The interview guide underwent a review by the PROTECT Community Advisory Board (CAB) members and was piloted among JHU staff who were not involved with PROTECT but identified as members of Indigenous communities. The feedback obtained from the CAB review and pilot testing allowed us to ensure that the interview guide was culturally appropriate and comprehensible for participants. For example, we modified one question to include relevant cultural context: “*What are some cultural beliefs in your community about vaccines? For example, some flu vaccines contain an inactive, sometimes called “dead,” flu virus. Culturally, this phrasing is worrisome to some people. What are your thoughts?*”.

The interviews were conducted by K.P. and E.P., who have over 4 years of experience as research program assistants and are members of the WMAT. Since the launch of the PROTECT study on 8 March 2021, K.P. and E.P. have worked with a subset of PROTECT participants as independent evaluators to conduct study assessments, establishing relationships and building rapport with the participants. K.P. and E.P. received training from JHU faculty members who specialize in qualitative research. The interviews took place outside of the participants’ homes. As part of the COVID-19 safety measures, all interviewers and study participants were required to wear masks and maintain six feet. In addition to formal training on how to use the interview guide, the interviewers also participated in role-play sessions that were observed by study coordinators. The coordinators provided additional training as needed. All interviews were conducted in English, lasted between 30 min and 1 h, and were audio recorded. The audio recordings were transcribed verbatim by a professional transcription company approved by our tribal partners. To maintain the anonymity of the participants, no personal details were included. Instead, alpha-numeric codes were utilized to assign data to each participant.

### 2.5. Data Analysis

The analysis process involved five steps. The first step began with H.O. and M.P. thoroughly reading all interview transcripts and writing memos to become familiar with the data. Preliminary findings were discussed during a research team meeting with M.C. and L.T., leading to a decision to employ qualitative content analysis methods [20] with an inductive approach [21] for data analysis. For the second step, H.O. randomly selected three interview transcripts and used an inductive approach to generate 47 initial codes such as access, confusing eligibility rules, discouraging stories, and protecting oneself. H.O. and M.P. reviewed the codes to ensure they accurately represented the observed data. For example, during this review, the code “exposure” was split into “not exposed” and “getting exposed at testing site” to better align with the data. This process was repeated for several codes until 61 codes were generated. Moving on to the third step, H.O. used the initial codes to develop a preliminary codebook including definitions of each code and how they would be applied to the data. The codebook was presented to M.P., K.P., E.P., M.C. and L.T. who provided feedback and approval. H.O. and M.P. then double-coded a randomly selected transcript to ensure consistent application of the codebook. Subsequently, H.O. and M.P. individually coded nine additional transcripts using the final codebook. The fourth step of the analysis involved organizing the coded data into six major categories informed by the interview guide and grouping of similar ideas. The categories, described below, include sources of COVID-19 information, COVID-19 care-seeking, social distancing, mask-wearing, COVID-19 testing, and COVID-19 vaccination. Barriers and facilitators within each category were identified, and a coding summary form with representative quotes was developed to illustrate the identified categories. This process informed the descriptive approach we took in organizing our results according to the participants’ experiences and perspectives within each of the identified categories. In the final step of the analysis, the results were reviewed to ensure consistency with the study aims and analysis objectives. We compared our findings with those from the motivational interviewing intervention conducted with a subset of PROTECT participants, thereby employing triangulation to verify the accuracy of the information we gathered during the analysis.

## 3. Results

### 3.1. Overview

In this section, we first lay out the demographics of the interview participants, we then present the qualitative findings organized into the six major categories. Representative quotes from participants have been grouped under each category to provide insights into participants’ perspectives and experiences. Later, in the discussion, we synthesize these findings into cross-cutting themes that can be applied in addressing future public health crises.

### 3.2. Demographics

Nineteen individuals participated in the qualitative interviews: 68.4% identified as female, and the age of participants ranged from 18 to 59 years with a median of 26. The majority had completed high school or higher education (57.9% completed high school or obtained a GED; 21.1% attended some high school; 15.8% completed some college/technical/vocational program; 5.3% held an associate’s degree). Regarding employment status, 36.8% were employed, while 57.9% were unemployed, and 5.3% did not specify. Marital status distribution revealed that 36.8% were single, 26.3% were legally married, 10.5% were cohabitating, 10.5% were separated, and 15.8% did not specify.

#### 3.2.1. COVID-19 Information

Participants obtained COVID-19 information from a variety of sources, including radio, television, pamphlets, online search engines, websites, social media, and family members. Common sources cited included Cable News Network (CNN), The Centers for Disease Control and Prevention (CDC), The United States Food and Drug Administration (FDA), The Indian Health Service (IHS), Johns Hopkins University, local clinics, and social media. However, the level of trust in these sources varied significantly. Several participants emphasized the reliability of information from family members working in healthcare settings. For example, one young adult female participant shared: “*I mostly go to my family that works in healthcare. They should know more… I wouldn’t ask my neighbors.*”. In contrast, social media was generally viewed with skepticism. Participants expressed concern about misinformation, noting that social media was not influential in their decisions regarding COVID-19 testing or vaccination. One participant said, “*The media lies a lot. I’d rather ask healthcare workers how it (the vaccine) works.*”. Others echoed similar sentiments, stressing the importance of independent thinking and seeking information from trusted sources rather than relying on social media. Despite the accessibility of information, participants made it clear that trust in the source was a critical factor in determining whether they acted on the information provided.

#### 3.2.2. COVID-19 Care-Seeking

When asked about seeking care when experiencing COVID-19 symptoms, participants had varying approaches. Some expressed the intention to seek care immediately at a health facility when experiencing symptoms, while others preferred to practice self-care until the individual’s condition became more severe. Participants also mentioned that body signals and the desire to feel better and have reassurance also motivated them to seek care. Several participants mentioned that factors motivating them to seek care when experiencing symptoms of COVID-19 included treating severe illness, preventing the spread of symptoms in households and communities, and protecting loved ones. As a female young adult described, “*All the symptoms that relate to the COVID and having to do with COVID and, just having those, I get worried. Just a little cough, just a little fever, and just some little side effect, and I would get scared, and I would want to go and get checked. I think (I would) go, go right away before it gets any worse. The main concern for me is just the virus and my daughter. Just keeping everybody safe, myself safe as well and just mainly my daughter.*” In terms of barriers to care seeking, some participants mentioned logistical factors such as lack of gas, transportation, and childcare as factors that might prevent them from seeking care when experiencing COVID-19 symptoms. Other participants reported the need to avoid exposure to COVID-19 in the process of seeking care as a factor that influenced an individual’s decision to not seek care. A female elder stated that she would only seek care if her symptoms became worse; if not, she would “*just stay home and take care of what symptoms I’m feeling. I do not recommend myself to go in because, um, the numbers are going up, and I don’t want to expose myself more to the dangers of it (COVID-19)*”.

#### 3.2.3. Social Distancing

We found that while most people had a clear understanding of social distancing, its implementation could be challenging due to various factors. One common obstacle was the perception that others do not adhere to social distancing guidelines, leading individuals to feel the need for extra caution, such as carrying hand sanitizers and multiple masks. As a young female participant described, “*I’m still doing everything that I was doing back in the city I’m still doing here. It’s just other people aren’t doing it, so I have to be like extra cautious I have to constantly have hand sanitizer with me. I never had to worry about carrying extra masks with me, but now I have to. People aren’t following the rules and they aren’t listening, so I think that’s what has me carrying more masks and just being more careful.*” Essential activities like shopping were mentioned as a reason for compromising on social distance. In addition, some participants expressed the emotional difficulty of being separated from loved ones for an extended period, resulting in feelings of loneliness. A female elder discussed the difficulties of social distancing, highlighting how being separated from her loved ones can evoke feelings of loneliness. “*It’s going on two years, and I haven’t even had a party or dinner. It’s been like this, I think I’m going to get used to this being a lonely grandmother, but they said they’re concerned about my health more than later on and that everything’s going to be back hopefully to normal. And so, we’ll start but we are not the one that is doing parties yet. We are not the ones that are doing gatherings yet. So, I’m a lonely grandma out here*”. Overall, these findings highlight the complexities and personal sacrifices associated with practicing social distancing during the ongoing pandemic.

#### 3.2.4. Mask Wearing

The topic of mask-wearing evoked strong opinions among the participants, revealing a range of perspectives. While some participants consistently adhered to COVID-19 guidelines by wearing masks, others expressed discomfort, and a few viewed masks as unnecessary after becoming vaccinated. Notably, some participants demonstrated unwavering commitment to following guidelines by always wearing masks, even if uncomfortable, and ensured their children did the same. For example, a female elder emphasized her dedication to mask-wearing, both for herself and her family: “*I wear my mask more. I still wear a mask out there even though I see a lot of people don’t, and I talk to my family in here, and talk to my older kids out there. I tell them you need to be careful with your surroundings. You need to wear your mask at all times. When I see them not wearing masks, I say, (gasps) where is your mask? They say okay yeah mom’s just all about masks. We have to keep practicing everything we were taught until we’re out of the woods. I know it’s hard to breathe. But that’s the thing we were educated on last year at the beginning of the pandemic, I still practice it*.” In contrast, some participants did not feel the need to continue wearing masks after becoming vaccinated. They expressed a sense of safety and comfort in going without masks in public, especially in certain environments. One young adult female participant shared: “*I hate it, (face masks). But I think ever since I got my vaccine, I consider myself safe. I hardly wear face masks out in public now, but I don’t have any reaction or like, the COVID symptoms. After I got the shot, I was worried about going to Florida on my trip. But once I was out there, no one wore a mask, and no one was social distancing at all. And there were like times it’s so humid there, and it doesn’t feel comfortable with a mask on, so I walked around without a mask as well. I didn’t want to, but it was like, easier to breathe*”. This perspective illustrates how, for some participants, the vaccine provided a sense of protection that reduced the perceived necessity of mask-wearing, particularly in environments where others were also unmasked.

#### 3.2.5. COVID-19 Testing

We found that several participants expressed reluctance to undergo COVID-19 testing as a preventive measure against the spread of the virus. A variety of factors contributed to this hesitancy. Notably, concerns regarding confidentiality emerged as a common theme, with participants citing it as a potential barrier to testing. Additionally, one participant highlighted discomfort with being tested at a local hospital due to the presence of family members who worked there. Among participants who reported a low likelihood obtaining testing, the majority justified this decision by stating that they had not been exposed to COVID-19 and did not experience any symptoms associated with the virus. Furthermore, some participants expressed apprehension about potential exposure at testing locations and discomfort associated with nasal swabs. A female young adult suggested that a more convenient, confidential alternative such as home test kits that use swabs collected from the front of the nose could potentially encourage more individuals to obtain testing. She said: “*Is there an easier way to do it (get tested) other than that big swab in the nose? The majority of the time, here on our reservation, we only get the swab. Well, that (referring to the home test kit) would definitely make it easier for me to get tested if it wasn’t that uncomfortable swab.*”

Our study revealed several factors that motivated participants to undergo testing. One prominent facilitator mentioned by participants was the accessibility of test centers, particularly the presence of mobile testing units in this community. Participants acknowledged that these mobile units made testing more convenient and easier to access. A female young adult expressed a desire for regular testing opportunities, suggesting the idea of designated testing centers where individuals could visit at regular intervals, regardless of experiencing symptoms or potential exposure to the virus. However, she also acknowledged the potential risk of overcrowding and increased transmission if such centers were not carefully managed. As the she described: “*It would be nice if there was a place where people could go every other week to go get tested instead of going (only) when you have a symptom, or when you feel that you ran into somebody that has that (COVID-19). It would be nice to just have a place to go where you can go on your own every other week, every other day, or just whenever, but then again that’s not safe too because everybody is going to be going and, and that’s how the virus starts to spread because most people don’t even wear a mask until you tell them. But that would be nice to have a little area set up like that for people, especially for work as well*”.

Additionally, participants indicated a willingness to receive testing when required by the workplace, school, or other authorities. One participant expressed a retrospective realization of the value of mandatory testing in places participants visit. According to this female young adult: “*Because I don’t have a job that requires it and I’m not sure if the place I’m going to requires one before you go there, which I wish they kind of require now that I think of it.*” Furthermore, participants mentioned the motivation to protect loved ones or to rule out COVID-19 when experiencing symptoms or potential exposure. This highlights the role of personal responsibility and concern for the well-being of others as driving factors for testing.

#### 3.2.6. COVID-19 Vaccination

Discouraging stories and misinformation about the COVID-19 vaccine emerged as significant barriers to vaccination among our participants. These stories primarily revolved around conspiracy theories, concerns about vaccination side effects, and uncertainties regarding vaccine effectiveness. A female elder shared a negative experience relayed by a friend who received the vaccine and reported experiencing severe symptoms, including the loss of smell. This anecdote contributed to the participant’s hesitation and highlighted the impact of personal accounts on vaccine perceptions. She shared: “*I went through a lot of stuff, and I don’t really want to go through these symptoms. And particularly, yesterday, I sat with a friend because she wanted to know how I was doing, so she goes, “I got vaccinated. I wish I never did, so I didn’t get the second shot. It got me so sick; I wish I never got the shot. I lost my [sense of] smell”. She said for three days, she went through the sickness. And she got scared. So that was one of the negative feedback that I got*”.

Conspiracy theories played a role in some participants’ decision-making process, as described by a female young adult. She mentioned YouTube videos and misinformation that propagated the idea of the government using the vaccine to monitor individuals through microchips. While she ultimately dismissed these theories, she acknowledged the initial influence these theories had on vaccination hesitancy. She said: “*Because like, everywhere else I looked at, like, YouTube videos, those were the main ones that kept talking about how the government was just trying to trick us into getting it so they can keep track of us… That it wasn’t safe, and that it was just a way for the government to keep track of everyone. I did hear that a lot before I got my shot. That was one of the reasons why I didn’t really want to get it, but then I thought about it, and how they said that there was like, small microchips in there. I don’t see how that’s possible*”.

Participants also highlighted lack of trust in the vaccine development process as a significant barrier. Some participants questioned the speed at which the vaccine was developed and expressed skepticism about why similar breakthroughs had not been achieved for other health issues. According to a female elder: “*They came out with the vaccination so fast, no, not years and years of testing. They just came up with it so fast, and there’s now like three vaccinations that we can get, so what I, what I personally shared with my family is if this is something they can come up with so quickly, why can’t they come up with something deadly to prevent us from getting cancer and, uh, tumors and all these things and some, this…But why was this vaccination so quick?*”.

Information gaps surrounding the vaccine’s content and effectiveness were reported by multiple participants. One participant shared concern about potential harm to pregnant individuals and babies based on stories they had heard. A female young adult referred to misinformation about one of the vaccines containing carcinogenic substances. She said: “*When the moms that were pregnant got it (the vaccine), they would die from it. It will kill their baby, and then it will kill them. So then, when I was pregnant, the doctor tried to ensure me that I wasn’t going to die. But I was just scared because I didn’t want to lose my baby. So, that’s why I never got the COVID vaccine when I was pregnant*”. Additionally, participants expressed a desire for clearer information about the vaccine, including its composition, potential side effects, and its impact on breastfeeding and children’s health. Several participants felt that insufficient information was provided during the vaccination process, leading to unanswered questions and hesitancy. A female young adult expressed: “*It felt like it wasn’t really enough information because they didn’t really tell us how the vaccine worked and what the side effects were going to be. They just listed the time and date, and where to go, and when to be there. And didn’t really tell us of how it can affect others or what’s all in the vaccine. I’m not sure if they gave out papers or flyers when they were giving out the shots, but on Facebook, I didn’t see any of that*”.

Complex eligibility criteria for COVID-19 vaccines raised concerns about equity and were a barrier to vaccination. Some participants struggled with the decision to receive vaccination when their children were not eligible for vaccination, raising concerns about potential differential health outcomes within participants’ families. A female young adult shared that “*I’m really thinking about it. For my daughter, for her to be safe but then again, I don’t think it’s fair that I get a shot and my daughter doesn’t. And, what if she gets sick and she gets a lot worse than me because I got the shot, and she didn’t get it? Like I… That’s another reason why I haven’t gotten it’s because she can’t get it, but it’ll be nice for me to get it so I wouldn’t have to bring back all that germs and all that stuff to her when I come back from work. This is, a really hard decision to make, but for her safety and mine, and everybody else around me. So most likely, I probably would get it. Will there be shots for, little kids six and under? No, six and over. Like, how would that affect them? Why aren’t they getting it? Why, why haven’t they come up with the shot for the little babies and all that?*”.

Several participants shared motivations for becoming vaccinated, which primarily centered around protecting themselves and their loved ones. One participant mentioned babysitting for relatives, indicating a responsibility to ensure the safety of the people they cared for. A female young adult expressed concerns about unvaccinated individuals being in close contact with their grandmother, who was at higher risk. She said: “*Well, because my grandmother, you know? My family, the unvaccinated ones, like, we had scares with them. Because they were positive, and they were around her (the grandmother). And then I babysit for my cousin, too. So, like, for them, you know? For them to make sure they’re safe. And then, like, what if I’m carrying it and I don’t know it, you know, and then I give it to them because I didn’t get sick, but they will?*” In some cases, participants mentioned specific events or trips as motivations for becoming vaccinated. This illustrates how the desire to engage in social activities and experiences free from COVID-19 concerns can motivate vaccination. A female young adult emphasized that the vaccine was a prerequisite for participating in the planned activity and said: “*I think the main reason that I got the vaccine was just to go on my Florida trip. And on top of that, after doing my research, it kind of just made me get it just for the girls here*”. Financial incentives provided by the tribal authorities also played a role in participants’ decision to become vaccinated. One participant mentioned the monetary benefits associated with vaccination, highlighting how the financial assistance supported their family. This incentive served as an additional motivator to prioritize health and prevent future illness. Finally, participants noted that the vaccination status of individuals in their household or community influenced their decision. One participant mentioned being encouraged by observing household members receiving the vaccine without experiencing negative consequences. This social influence played a role in the participant’s willingness to become vaccinated as well.

## 4. Discussion

In this qualitative study, we explored factors that influence COVID-19 testing and vaccine uptake among White Mountain Apache Tribal members to inform testing and vaccine communication campaigns and logistical support promoting testing and vaccination. The COVID-19 landscape changed rapidly during this study and looks even more different now, with the Biden administration ending the national emergency response to the pandemic in April 2023 [22]. However, we still have an opportunity to optimize public health strategies for the prevention of COVID-19 and to prepare for future outbreaks. The following key findings from this qualitative study reveal insights into the experiences of the pandemic in Indigenous communities and can point us towards strategies to better address infectious diseases going forward, with a specific lens of health equity.

These findings from an Indigenous community share some similarities and highlight some differences from other non-Indigenous samples. Our study not only examined barriers and facilitators to testing and vaccine uptake but also gathered data around mask-wearing, social distancing, care-seeking, and COVID-19 information. A large study of a nationwide sample of *n* = 536 youth also investigated opinions about how to increase COVID-19 vaccine uptake and found that the top factors cited were more testing of vaccines and safety data, incentives, education about the vaccines, you or someone getting COVID-19, and mandates [23]. Other motivators that were less frequently endorsed included social pressure or support, access, recommendation from a trusted source, trust in government, science, and healthcare, permission from a family member, and other means of taking the vaccine, such as a nasal mist. A large cohort study of participants from Hong Kong and Singapore found that 75% of barriers to becoming vaccinated were explained by mistrust in health authorities, low confidence in the vaccine, misinformation and political views [24]. While the information that emerged from our study has much in common with these samples, the emphasis or weight of social and collective facilitators is worth noting in our sample. A study in Sub-Saharan Africa also noted these types of factors, including protecting others in their country and perceptions of social norms as reasons for vaccine take-up [25]. This cross-cutting theme will be discussed more in our synthesis below.

An important lesson from our findings is the power of storytelling and personal experiences in shaping public health efforts. Personal anecdotes played a significant role in shaping perceptions not only about COVID-19 itself but also about the testing and vaccination processes. Interestingly, these stories were shared both in-person and through social media platforms, which have become major avenues for storytelling. In a qualitative study of COVID-19 vaccine decision-making among urban Native Americans, word of mouth and seeing role models being vaccinated were recognized as vital communication among Indigenous samples [26,27]. However, concerns about potential misinformation from personal anecdotes were also expressed. Therefore, we propose leveraging the power of positive experiences and personal narratives to combat the prevailing negative stories. By amplifying the voices of individuals who have had positive experiences with testing and vaccination, public health can counteract the discouraging narratives and promote behavioral changes within communities. Online platforms are used not only for sharing cultural practices like cooking, gardening, and drumming but also for engaging in public health discussions and disseminating community-wide information [12]. Several studies have highlighted the use of social media platforms for COVID-19 education targeting Indigenous communities [27,28,29,30].

Proactive community members also sought credible information from trusted sources and became advocates, encouraging others to receive testing and vaccination, playing a pivotal role in preventing the spread of COVID-19. Despite a context of potential medical mistrust that results from the pervasive mistreatment and discrimination that people of color experience in the healthcare system [31], participants trusted healthcare workers to provide them with reliable information, particularly Indigenous providers and those from the Indian Health Service (IHS) who serve as the primary care providers for Indigenous communities [27,32]. Mistrust and vaccine hesitancy were widely acknowledged as a challenge to COVID-19 vaccine uptake for Black and Latinx communities and among at-risk sub-populations (e.g., those with HIV and/or substance misuse) [33,34,35], but less so for Indigenous communities, who led the nation in the percentage of vaccinated individuals in the months following the roll-out of COVID-19 vaccines [36]. From a nationally representative survey in Mexico, Indigenous language speakers who were unvaccinated were more likely to cite negative beliefs about the vaccine or fear as reasons for not being vaccinated [37]. The division between science and society has presented a need for credible messengers and strategic public health communication, as well as equipping both public health professionals and community members to identify and address misconceptions, ensuring that individuals receive accurate information to make informed decisions regarding COVID-19 prevention measures.

Furthermore, our study revealed common questions and doubts that could have been addressed more thoroughly and earlier in the pandemic to foster trust and confidence. Several participants described the news as “lies,” which may have resulted from conflicting advice as scientists learned more about the virus. This environment created an opportunity for conspiracy theories to gain traction. Search engines like Google were frequently cited as the primary source of information for participants. To address this issue, it is crucial to promote up-to-date public health organizations and websites to appear as top search results [38]. Similar strategies should be employed to promote verified public health information on social media platforms, considering that Facebook was identified in this study as another common source of information. Implementing online fact-checking initiatives and investing in dissemination funds could also prove beneficial in safeguarding people from the harms of misinformation. Another potential strategy to address the overwhelming and often contradictory deluge of information is tailored messaging [39], in which public health practitioners focus on understanding what community members want to know about public health problems and solutions. Like the positive interpersonal interactions observed earlier, individuals tend to trust information more when they feel heard, and their questions are answered thoughtfully.

Another robust theme emerging from this study is that a strong sense of social responsibility in the White Mountain Apache community motivated adherence to prevention recommendations—most themes indicated that participants were willing to engage in protective behaviors for oneself to protect loved ones, especially Elders, and the community, which also emerged in the study by Labbe and colleagues. This finding is in line with several other studies in non-Native specific [40,41,42,43] samples that have found that public health messaging that uses a moral frame focused on the responsibility of protecting others, including family and friends, is a more effective approach for influencing behaviors to mitigate COVID-19 transmission than messaging that emphasizes personal benefits [41] or fear-based messaging [42]. In another qualitative study [43] drawn from a random panel of 3000 phone numbers for adults > 18 years of age across Alberta, Canada, participants discussed the importance of staying home when ill to protect others, that a vaccine could be used to protect oneself and others, and that the perceived threat to the community, especially those with pre-existing conditions or who were immunocompromised, was motivation to wear a face mask in public (also, Carpraro and Barcelo [44]). This same study found; however, that other participants said they would not take a COVID-19 vaccine, feeling that COVID-19 would not affect the health of participants’ family members, which is not surprising given that there can be wide variability in individuals’ COVID-19 attitudes, beliefs, and behaviors. Among an urban Indigenous sample of over 1400 survey participants, respondents endorsed the responsibility to protect the Indigenous community and culture as the main motivators for becoming vaccinated [45]. Indigenous vaccination campaigns tapped into these strengths (e.g., https://forourpeople.uihi.org (accessed on 22 February 2024) and https://caih.jhu.edu/resource-library (accessed on 22 February 2024). In addition, Indigenous communities used collective action, often with tribal mandates, enacted through the power of tribal sovereignty, to promote other individual protective behaviors including shelter in place, curfews, and social distancing orders over longer periods of time than seen in other communities [46]. This strong sense of social responsibility might be attributable to a sense of solidarity and connectedness to all beings and creation, as well as respect for Elders and culture-bearers who were vulnerable to COVID-19 [28].

## 5. Strengths and Limitations

Our study showcased several notable strengths. First, we were able to utilize face-to-face interactions by implementing essential safety measures, such as masks and distancing, which may have allowed us to establish a deeper rapport with participants. This approach potentially enhanced the richness of the data collected, as in-person interviews might have offered a unique advantage in accessing nuanced non-verbal cues that could have been missed in virtual settings. In addition, the design, implementation, and data collection for this study involved trusted members of the community, who are more likely to have an intrinsic understanding of local nuances and social norms. This familiarity not only likely enhanced the quality of interactions but also potentially enriched the data collected. In our experience in the study communities, participants often exhibited increased comfort when sharing information with someone from the community, which could lead to more comprehensive data. These community-based interviewers provide an invaluable ‘insider perspective’, potentially unearthing and examining areas of interest or concern pertinent to the community that might otherwise be overlooked by external researchers. With community members as research team members, our understanding of participants’ perspectives and experiences was potentially enriched, likely leading to a more comprehensive analysis of our findings.

We acknowledge certain limitations in our qualitative study. Despite our efforts to ensure participants’ comfort, the pandemic might have induced hesitancy or unease during in-person interviews. Interviewers sometimes exercised caution in probing further to minimize participants’ discomfort, especially considering the unique challenges of conducting research during such unprecedented times. While we made attempts to minimize bias through coding agreement and research group discussions, the inability to seek direct clarifications from participants could have introduced inaccuracies in accurately reflecting participants’ interview responses during the coding process. Additionally, less probing may have resulted in less detailed and richer responses in response to questions. Furthermore, we enrolled primarily young and middle-aged adults in a single tribal community, so the findings of our study may not be generalizable. Despite our intention to include participants from all three PROTECT sites, we were unable to do so. From the parent study, only 64 participants were enrolled due to the significant challenges of conducting a research study during an evolving pandemic and the needed changes to the research protocol. The Navajo sites had an extra layer of IRB approval; therefore, it took longer for these sites to recruit their participants, and the main sample we drew had more WMAT participants. Additionally, the parent PROTECT study, from which all qualitative sub-study participants were derived, excluded individuals who had received the COVID-19 vaccine, so these findings may represent a subset of individuals who may have initially been vaccine-hesitant.

Nonetheless, the findings from our sample size of *n* = 19 participants align with other qualitative studies and offer valuable insights into the unique reasons behind vaccine hesitancy within this specific population. Moreover, the study’s focus on exploring factors contributing to testing and vaccine hesitancy in this group provides significant programmatic opportunities for the future. By understanding participants’ reasons for obtaining or avoiding vaccines, public health initiatives can effectively address concerns and develop targeted strategies to promote testing and vaccine acceptance within Indigenous communities.

## 6. Conclusions

This study sheds light on the factors influencing COVID-19 testing and vaccine uptake among Indigenous adults, guiding testing and vaccine communication campaigns and logistical support for vaccination promotion. Storytelling and personal experiences emerged as significant influences, shaping perceptions about COVID-19 testing and vaccination. Discouraging stories and misinformation, both in-person and through social media, contributed to testing and vaccine hesitancy. Proactive individuals seeking credible information played a crucial role in preventing the spread of COVID-19. Tailored messaging and promoting reliable sources offer potential strategies to combat misinformation. The strength of social responsibility and collectivism in Indigenous communities facilitated protective behaviors. These findings emphasize the importance of community-driven public health efforts and collective action in managing pandemics within Indigenous communities. Collaborative efforts with communities will play a key role in building a healthier and more resilient future for all.

## Data Availability

The data for this study are presented throughout this manuscript as de-identified participant quotes. Further data requests are not publicly available due to tribal sovereignty and ownership of the data by participating tribes.
